# Impact of sprint interval training on post-fatigue mitochondrial rate in professional boxers

**DOI:** 10.1007/s00421-024-05594-0

**Published:** 2024-09-03

**Authors:** Andrew Usher, John Babraj

**Affiliations:** Dept of Sport and Exercise Science, Abertay University, Bell St, Dundee, DD1 1HG Scotland

**Keywords:** NIRS, Rectus femoris, Oxygenation, Desaturation, Recovery, Mitochondria

## Abstract

**Purpose:**

Professional boxing is a sport that requires a high aerobic capacity to prevent fatigue and allow athletes to perform over 4–12 rounds. Typically, athletes will go into a heavy training period in a pre-bout camp lasting 6 to 9 weeks. This study investigates the impact of 3 weeks of repeated Wingate sprint interval training, performed on standard gym ergometer bikes, on skeletal muscle endurance and mitochondrial function.

**Methods:**

Ten male professional boxers (age: 26 ± 4 years, height: 175 ± 5 cm, weight: 70 ± 5 kg) participated in the study. Baseline testing involved a NIRS monitor attached to the rectus femoris muscle prior to an incremental time to exhaustion test on a treadmill. After the treadmill test participants underwent a series of arterial occlusions to determine mitochondrial function post-volitional exhaustion. Participants then continued their own training for 3 weeks and then repeated baseline testing. After the second testing session, participants undertook three weekly sprint sessions consisting of 3 × 30 s maximal sprints with 60 s recovery. Testing was repeated 3 weeks later.

**Results:**

The time to exhaustion increased by > 6% after 3 weeks of sprint interval training as compared to baseline and control (*p* < 0.05). Skeletal muscle oxygen saturation (SmO_2_) at exhaustion was increased by 5.5% after 3 weeks of sprint interval training as compared to baseline and control (*p* = 0.008). Skeletal muscle mitochondrial rate post exhaustion was increased by 160% after 3 weeks of sprint interval training as compared to baseline and control (*p* < 0.001).

**Conclusion:**

The study demonstrated that SIT led to increased incremental time to exhaustion, higher SmO_2_ levels at volitional exhaustion and increased mitochondrial rates in professional boxers. These findings suggest that SIT should be an integral part of a boxe’s conditioning regimen to improve performance and safety within the ring.

**Supplementary Information:**

The online version contains supplementary material available at 10.1007/s00421-024-05594-0.

## Introduction

Amateur boxing is typically seen as a highly intermittent sport, a classification supported by studies like Nassib et al. (2017), which also notes the unique feature of one-minute recovery periods between rounds. The debate around the energetic costs of boxing, whether it leans more towards anaerobic (Ghosh AK, Goswami A 1995) or aerobic energy use (Davis et al. 2014), highlights the role of anaerobic energy production in the sport. However, professional boxing demonstrates a more intricate pattern of oxygen use and recovery, particularly in the rectus femoris muscle. Recent research (Usher and Babraj 2023) suggests a three-component model for skeletal muscle oxygenation and recovery, and consistent muscle activity as opposed to intermittency. This consistent activity suggests a continuous metabolic engagement of the mitochondria within the rectus femoris, maintaining a stable equilibrium between oxygen delivery and use throughout the rounds (Usher and Babraj 2023). The recovery from exercise has been shown to be an aerobic process (Zoll et al. 2006; Porter et al. 2015) and improved mitochondrial function will enhance recovery between rounds. Therefore, there is a need for appropriate training methods that enhance mitochondrial rate and function in professional boxing.

Sprint interval training (SIT) has been defined as short duration, typically 30 s or less, repeated supramaximal efforts (Gibala et al. 2012). Most studies utilising this approach use 7.5% of body mass to control intensity (Burgomaster et al. 2008; MacInnis and Gibala 2017) and has been shown to increase mitochondrial content and function within skeletal muscle after just 6 sessions, along with increases in glycolytic enzyme activity, buffering capacity and glycogen stores (Burgomaster et al. 2008; Little et al. 2010; Jacobs and Lundby 2013). To date the impact of SIT on mitochondrial function has been limited to biopsy pre- and post training and then measuring mitochondrial enzyme content and activity (Perry et al. 2010; MacInnis and Gibala 2017) and as such has not been explored in professional athletes. As well as changes in mitochondrial capacity, SIT has been shown to improve oxygen extraction into the skeletal muscle (Bailey et al. 2009) and increase mitochondrial oxygen affinity (Larsen et al. 2020) which will lead to improved energy production and utilisation (Booth and Thomason 1991; Mathuram et al. 2022) and can improve an athlete’s ability to sustain high-intensity efforts (Juel et al. 2004; Nielsen et al. 2017) and delay the onset of fatigue (Yamagishi and Babraj 2017).

The use of near infrared spectroscopy (NIRS) with rapid arterial occlusion (AO) have been used to noninvasively assess mitochondrial function post exercise (Ryan et al. 2013, 2014; Sumner et al. 2020; Hanna et al. 2021). Mitochondrial measurement with NIRS involves using a blood pressure cuff to create an AO, creating ischemic conditions in muscle tissue which separates the process of oxygen delivery and consumption (Maliszewski et al. 2024). Lagerwaard et al. (2019) reported a 40% faster mVO_2_ recovery in the gastrocnemius muscle of participants with a high endurance capacity as compared to low endurance capacity (Lagerwaard et al. 2019). Brizendine et al. (2013) showed an approximate twice the amount of mitochondrial capacity in the vastus lateralis muscle in endurance trained athletes as compared to inactive individuals (Brizendine et al. 2013). However, to date this approach has only been looked at during submaximal exercise efforts and has not been used in elite sport.

Given the short duration of training camps in professional boxing and the need to enhance muscle oxidative capacity then the use for a SIT based intervention may be beneficial. The efficiency and brief duration of these exercises make them easy to integrate into tight training schedules and would allow more time to technical components of the sport. Therefore, this study sought to determine the impact of a 3 week SIT intervention on mitochondrial capacity in trained professional boxers. We believe this study is the first to examine mitochondrial values with professional boxers as well as the first study to examine it following volitional exhaustion. We hypothesised that SIT would improve skeletal muscle fatiguability and improve post-exercise mitochondrial function in professional boxers.

## Methods

### Subjects

9 male professional boxers (age: 26 ± 4 years, height: 175 ± 5 cm, weight: 70 ± 5 kg body fat: 11 ± 3%, rectus femoris fat thickness: 4.6 ± 2.0 mm) were recruited for this study. Body composition remained unchanged throughout the study (*p* > 0.05). Each athlete held a current professional licence with the British Boxing Board of Control (bouts: 15 ± 5). Participants were excluded if there was a loss of training days over the last 3 months due to musculoskeletal injury or sanction from British Boxing Board of Control preventing the athlete from taking part in training or sparring. Participants were informed verbally and in writing on the risks and benefits of the study prior to signing informed consent form and was approved by Abertay University Research Ethics Committee (EMS5604). The study was carried out in line with the Declaration of Helsinki, except for the registration in a database.

### Testing

Participants were instructed to adhere to their regular training routines and dietary habits throughout the study duration. They were also advised to avoid intense physical activities and alcohol consumption for 24 h prior to each laboratory visit. Additionally, participants were required to be in a fasted state, having abstained from food or drink intake for at least 4 h prior to their arrival at the Human Performance Laboratory.

**Fig. 1:**
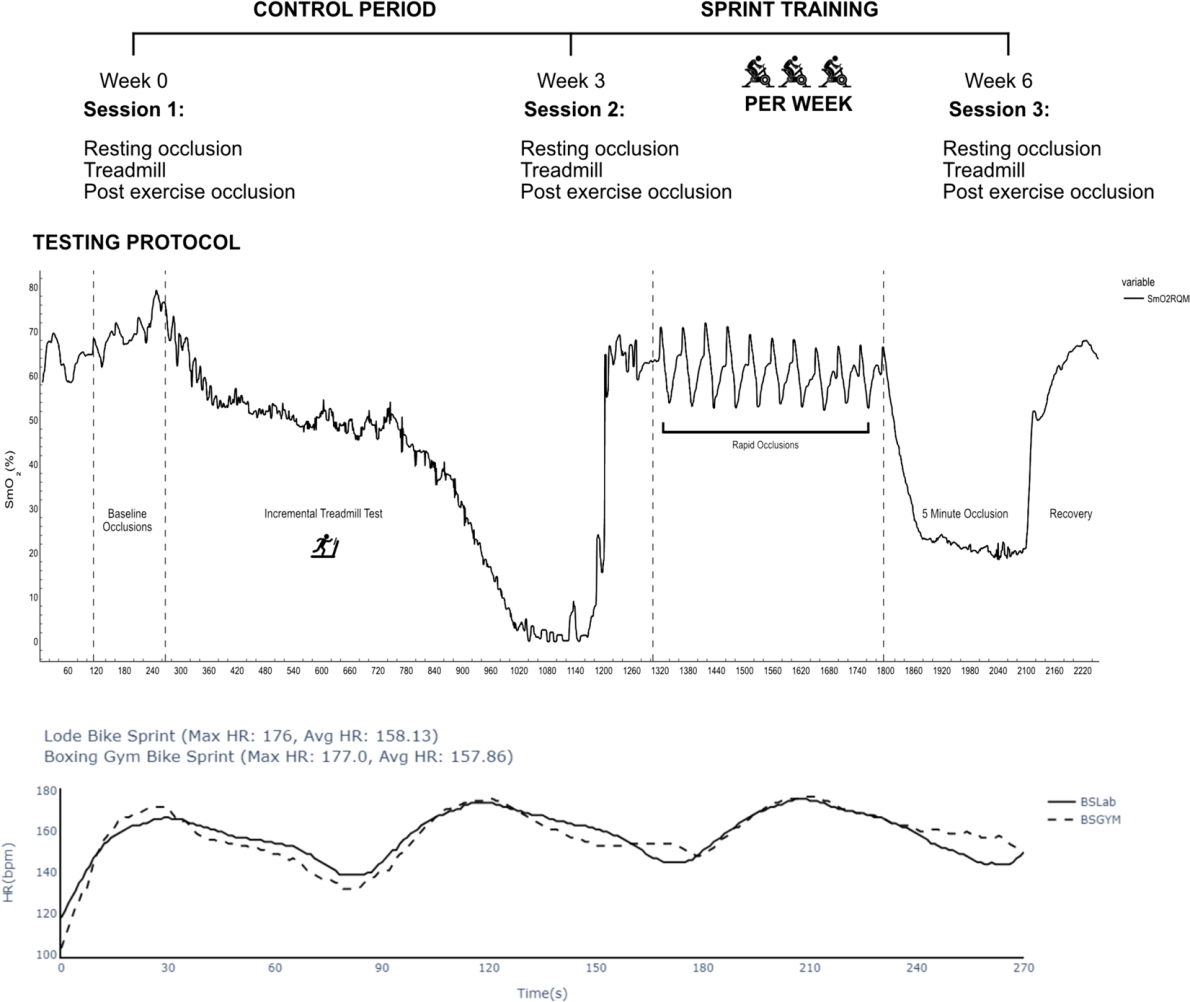
Occlusion protocol (right rectus femoris muscle), bike sprints (boxing gym, lode bike)

The research protocol comprised three distinct laboratory visits: an initial familiarisation session, a control period assessment, and a final evaluation post a 3 week sprint training intervention (Fig. 1). During each visit, participants had near infrared spectroscopy (NIRS; Moxy Monitor, Fortiori Design LLC, USA) taped to the rectus femoris muscle of the left and right leg. The Moxy is a lightweight (48 g) and small (62 × 52 × 15 mm) device that uses continuous four wavelengths (680 nm, 720 nm, 760 nm, and 800 nm) reporting values of SmO_2_ in the form of a 0–100 percentage scale (Feldmann et al. 2019).

The moxy monitor was set to high speed, 0.5 s update with no smoothing. The Moxy monitor has been shown to be valid and reliable during exercise with a greater dynamic scale than the Portamon monitor (McManus et al. 2018; Feldmann et al. 2019); and has been used to explore muscle oxygenation during a wide range of sporting environment and training domains (Paquette et al. 2018; Perrey and Ferrari 2018). Participants then attached a heart rate monitor (Polar H10, Polar Electro, UK). All data was then collected via Bluetooth transmission to the VO2 master app (VO2 Master Health Sensors Inc, Canada).

### Body composition

All participants had height determined by stadiometer (Seca 264, Seca, UK) and body composition was determined by bioimpedance (Tanita MC-780, Tanita, Japan). The fat thickness on the rectus femoris muscle was determined by ultrasound (EagleView, Wellue, Diamond Bar, CA, USA) as a fat thickness of greater than 14 mm will impede NIRS signal (Feldmann et al. 2019).

### Testing protocol

The occlusion protocol involved the application of a blood pressure cuff to the upper thigh, connected to a rapid inflation system, and the leg and was rapidly inflated to > 300 mmHg. The protocol (Fig. 1) included three rapid occlusions at rest in the supine position, each lasting 15 s with 30 s between each occlusion (see S1 for SmO_2_ during occlusion). Cuff inflation reaching 300 mmHg in under 2 s. The time from cuff inflation to decline in rectus femoris muscle oxygenation was 0.319 ± 0.114 s. Participants then completed an incremental treadmill test to volitional exhaustion, starting at a speed of 10 km.h^−1^ with a 0% incline, increasing by 1 km.h^−1^ every minute until reaching 18 km.h^−1^, after which incline increased by 1% every minute. Two minutes post-treadmill run, participants underwent a series of 10 rapid occlusions (15 s each with 30 s between each occlusion) followed by a single 5 min occlusion whilst in a supine position.

### Control period

Participants were then instructed to continue with their own training for the next 3 weeks after the first testing session before returning to the lab at the same time of day and repeating the testing procedure.

### Intervention

Following the second visit to the lab participants were given a familiarisation of 30 s effort against 7.5% body mass on the ergometer (LODE). Briefly, participants were instructed to bring the speed up to 85 rpm and then given a 3, 2, 1 countdown and instructed to cycle as fast as possible when hearing 1. Heart rate was recorded throughout, and athletes instructed that this is where the heart rate needs to be during the first sprint in subsequent training sessions. The intervention entailed participants performing 3 × 30 s maximal efforts with 60 s recovery on 3 days of the week for 3 weeks. Participants were instructed that ideally there should be 48 h between sprint sessions. Effort levels during these remote bike sprints were monitored using the Polar H10 heart rate data to ensure consistent exertion, with a self-reported adherence of 100% (Fig. 1).

### Data analysis

Heart rate and SmO_2_ were exported from VO2 master as a 1 s average and processed in Python Jupyter Lab (version 3.3.2). A median average 5 s filter was applied to the data to smooth any movement artefacts (Buchheit and Laursen, 2011). SmO_2_ was used for analysis as it gives a better indication of skeletal muscle oxygenation when blood flow is not steady (Buchheit and Ufland 2011). Correcting for blood volume changes during occlusions to accurately assess changes due to oxygen consumption is required as blood volume changes can obscure the true NIRS signals reflecting muscular oxygen dynamics (Ryan et al. 2012). To effectively correct these signals, the blood volume change must be proportioned between oxygenated and deoxygenated blood components. This differentiation is important because it allows for a more precise interpretation of the NIRS data, isolating the oxygen consumption component from the confounding effects of blood volume fluctuations (Ryan et al. 2012).

$$\beta \left( t \right) = \frac{{\left| {O_{2} Hb(t)} \right|}}{{\left( {\left| {O_{2} Hb(t)} \right| + \left| {HHb(t)} \right|} \right)}}$$

Post-exercise occlusion a linear regression model was used for partial curve fitting to calculate the rate of muscle oxygen consumption (mVO_2_) during arterial occlusion. Curve fitting was expressed via a monoexponential curve fit to determine mitochondrial rate function (Ryan et al 2012). SmO_2_ during the treadmill test was analysed using linear curve fitting from the start of the final desaturation period to plateau before exhaustion (Fig. 1) with the slope representing rate of fast desaturation and rate of fast resaturation taken as the slope from the linear part of post-exercise recovery (Fig. 1). Area under the SmO_2_ post-treadmill recovery curve was calculated using the trapezoidal method and reflects oxygen availability during the recovery phase.

### Statistical analysis

All data is presented as means ± standard deviation. Main and interaction effects are detailed, with post hoc pairwise analyses available in subsequent tables. Statistical analyses were conducted using Jamovi software (version 2.3.13). Normality checks were performed using the Shapiro–Wilks test, and all data were found to be normally distributed. Grubbs test for outliers was applied to the time to exhaustion and mitochondrial rate datasets, with an alpha value of 0.05. The G statistic for time to exhaustion was 1.959 and the critical value was 2.215, the G statistic for mitochondrial rate was 1.639 and the critical value was 2.651. Since the G statistic is lower than the critical value for both time to exhaustion and mitochondrial rate then there were no outliers within the dataset. All data was analysed using a repeated measures ANOVA for occlusions. Where there was a significant main effect then a least squares difference post hoc test was applied to determine where differences occurred. Significance was accepted at *p* < 0.05.

## Results

**Fig. 2:**
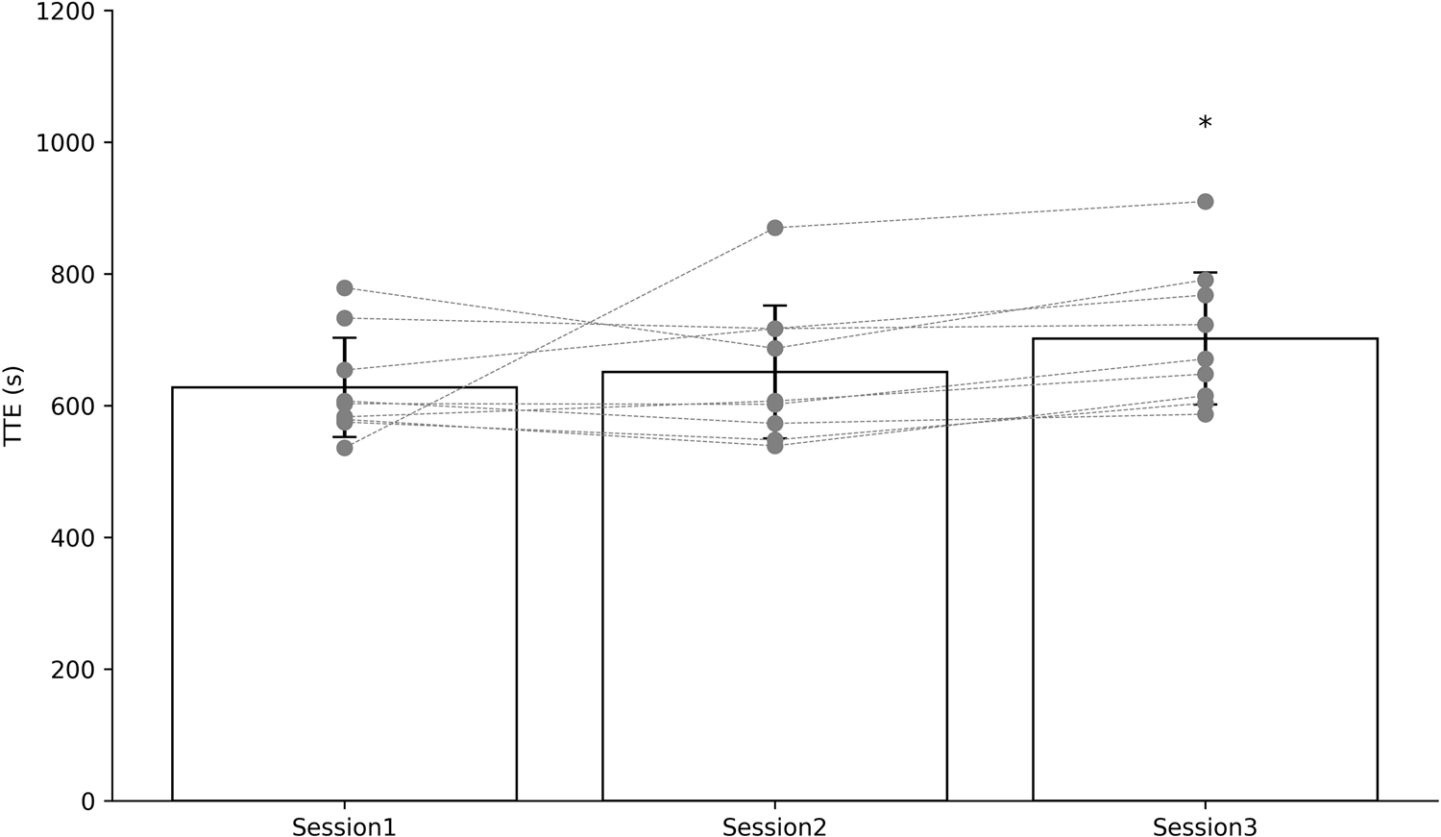
Incremental time to exhaustion, time to exhaustion across the 3 testing sessions. *p < 0.05 session 3 compared to session 1 or 2

There was a significant main effect for time in incremental time to exhaustion (*p* = 0.004, Fig. 2). Following the control period there was no significant difference in time to exhaustion between session 1 and 2 (session 1: 662 ± 100 s, session 2: 651 ± 107 s, *p* = 0.490, Fig. 2). There was a significant difference in time to exhaustion following the 3  week sprint interval training protocol between session 1 and 3 (session 1: 662 ± 100 s, session 3: 702 ± 106 s, *p* = 0.023, Fig. 2) and session 2 and 3 (session 2: 651 ± 107 s, session 3: 702 ± 106 s, *p* = 0.001, Fig. 2).

There was no significant difference in pre-exercise SmO_2_ (*p* = 0.153, Table 1), the change in SmO_2_ across the test (*p* = 0.804, Table 1) or the rate of fast desaturation at the end of the test (*p* = 0.268, Table 1, Fig. 1). There was a significant main effect for time (*p* = 0.008, Table 1) in the SmO_2_ value at exhaustion. There was no significant difference in the value of SmO_2_ at exhaustion between session 1 and session 2 (*p* = 0.773, Table 1). Following 3 weeks of sprint interval training there was a significant difference in SmO_2_ at exhaustion as compared to session 1 (*p* = 0.017, Table 1) or session 2 (*p* = 0.006, Table 1). There was a significant main effect for time (*p* = 0.037, Table 1) in the SmO_2_ post-treadmill recovery area under the curve. There was no significant difference in the value of AUC between session 1 and session 2 (*p* = 0.350, Table 1). Following 3 weeks of sprint interval training there was a significant difference in AUC as compared to session 1 (*p* = 0.020, Table 1) or session 2 (*p* = 0.050, Table 1).

**Table 1:** SmO_2_ across the incremental test

	Session 1	Session 2	Session 3
Pre SmO_2_ (%)	71.9 ± 11.9	75.1 ± 15.3	82.3 ± 8.8
Exhaustion SmO_2_ (%)	5.2 ± 5.6	5.9 ± 6.5	11.3 ± 6.0^a,b^
Absolute difference in SmO_2_ (%)	66.6 ± 12.2	69.2 ± 15.3	71.0 ± 12.2
Rate of fast desaturation (%.s^−1^)	−1.27 ± 0.67	−1.73 ± 1.05	−1.96 ± 0.69
Rate of fast resaturation (%.s^−1^)	2.29 ± 1.69	3.67 ± 2.68	2.46 ± 1.61
Recovery AUC (%.s)	3655 ± 1623	4837 ± 2537	6510 ± 2423^a,b^

^a^*p* < 0.05 session 3 compared to session 1

^b^*p* < 0.05 session 3 compared to session 2

**Fig. 3:**
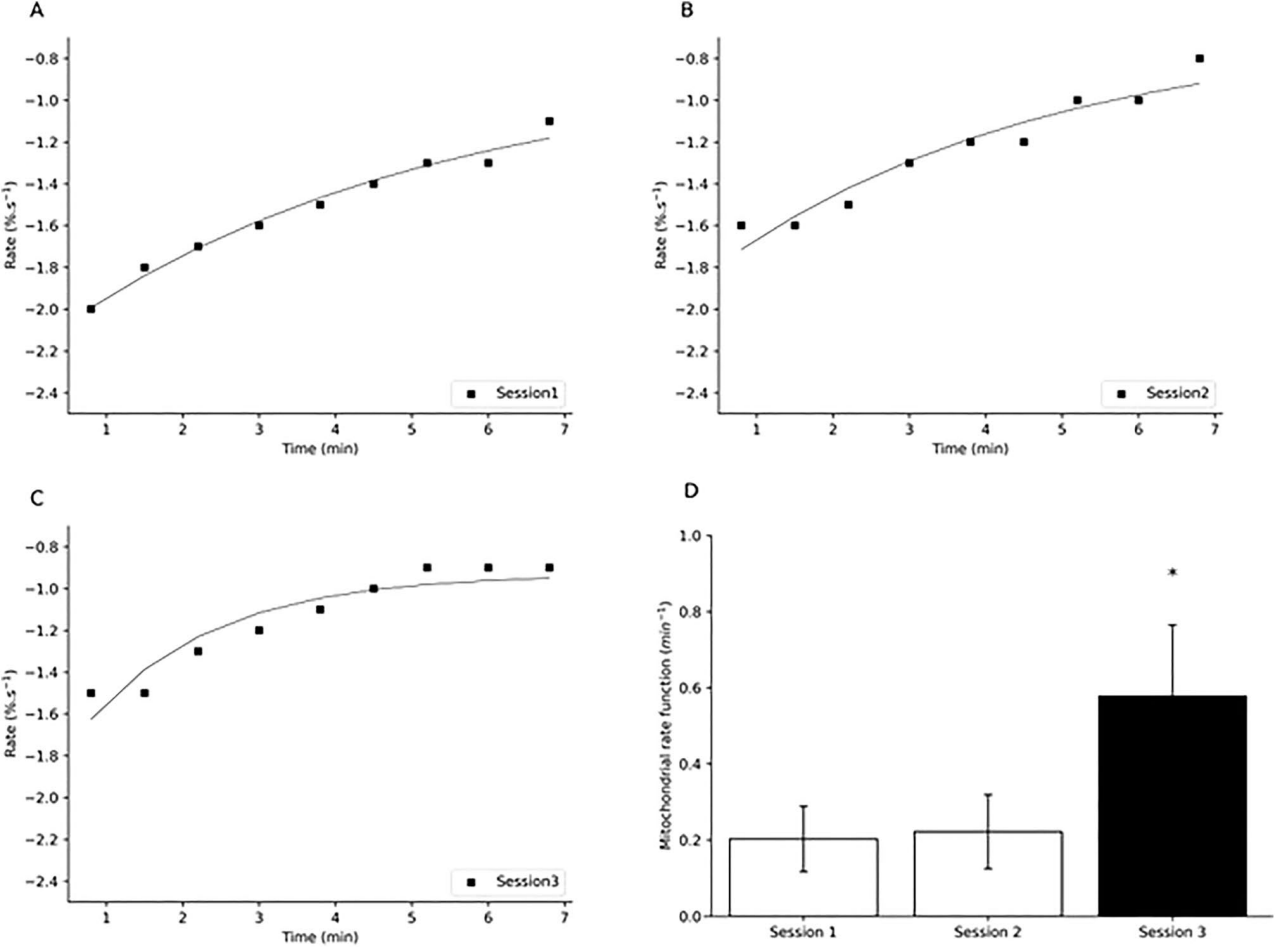
Mitochondrial function post-incremental treadmill test A: individual occlusion curve session 1 B: individual occlusion curve session 2 C: individual occlusion curve session 3 D: Mitochondrial rate function

Heart rate across the occlusion period was 104 ± 15 bpm in session 1, 109 ± 14 bpm in session 2, 108 ± 16 bpm in session 3, with no significant difference between mean values (*p* = 0.061) or standard deviation in heart rate across the occlusions (*p* = 0.161). Saturation in the exponential curve fit was only seen in session 3 post-sprint training but not in session 1 or session 2 (Fig. 3A–C). There was a significant main effect for time for mitochondrial rate function post-treadmill run to volitional exhaustion (*p* < 0.001, Fig. 3D). Following the control period there was no significant difference in mitochondrial rate function between session 1 and 2 (session 1: 0.203 ± 0.086 min^−1^, session 2: 0.222 ± 0.97 min^−1^, *p* = 0.422, Fig. 3D). There was a significant difference in mitochondrial rate function following the 3 week sprint interval training protocol between session 1 and 3 (session 1: 0.203 ± 0.086 min^−1^, session 3: 0.578 ± 0.187 min^−1^, *p* < 0.001, Fig. 3D) and session 2 and 3 (session 2: 0.222 ± 0.097 min^−1^, session 3: 0.578 ± 0.187 min^−1^, *p* < 0.001, Fig. 3D).

There was a significant occlusion effect for SmO_2_ fast resaturation rate (*p* = 0.029; Table 2) but no session effect (*p* = 0.139; Table 2) or session by occlusion effect (*p* = 0.992). In session 1, occlusion 3 was significantly different from occlusion 4, 5 and 10. In session 2, occlusion 10 was significantly different from occlusion 3, 4 and 5 and occlusion 3 was significantly different from occlusion 5.

**Table 2:** Rate of SmO_2_ desaturation and resaturation during post exercise occlusions

	Session 1	Session 2	Session 3
FDR			
Rest	−0.54 ± 0.09	−0.55 ± 0.18	−0.41 ± 0.11
Occlusion 1	−2.28 ± 0.47	−1.77 ± 0.69	−1.76 ± 1.05
Occlusion 2	−1.96 ± 0.34^a^	−1.60 ± 0.46	−1.53 ± 0.61
Occlusion 3	−1.81 ± 0.35^a,b^	−1.55 ± 0.44	−1.33 ± 0.63^a^
Occlusion 4	−1.71 ± 0.36^a,b,c^	−1.49 ± 0.26	−1.23 ± 0.54^a^
Occlusion 5	−1.64 ± 0.36^a,b,c^	−1.35 ± 0.47	−1.16 ± 0.37^*^
Occlusion 6	−1.51 ± 0.31^a,b,c,d,e^	−1.24 ± 0.34^a,b,d^	−1.06 ± 0.23^b,*^
Occlusion 7	−1.45 ± 0.33^a,b,c,d,e^	−1.19 ± 0.18^a,b,c,d,*^	−1.00 ± 0.29^a,b,*^
Occlusion 8	−1.36 ± 0.34^a,b,c,d,e,f,g^	−1.11 ± 0.29^a,b,c,d,*^	−0.95 ± 0.22^a,b,^^*^
Occlusion 9	−1.31 ± 0.33^a,b,c,d,e,f^	−0.98 ± 0.23^a,b,c,d,e,f,g,h,*^	−0.94 ± 0.34^a,b^^,*^
Occlusion 10	−1.14 ± 0.28^a,b,c,d,e,f,g,h,i^	−0.85 ± 0.21^a,b,c,d,e,f,g,h,i,^^*^	−0.94 ± 0.28^a,b^^,*^
FRR			
Rest	1.38 ± 0.73	1.35 ± 0.56	0.98 ± 0.44
Occlusion 1	3.75 ± 2.64	2.94 ± 1.57	3.25 ± 2.29
Occlusion 2	4.31 ± 3.59	2.84 ± 1.47	3.00 ± 2.64
Occlusion 3	4.37 ± 2.97	2.96 ± 1.73	2.72 ± 2.05
Occlusion 4	3.51 ± 2.42^c^	2.77 ± 1.58	2.43 ± 2.17
Occlusion 5	3.11 ± 2.10^c^	2.37 ± 1.58^c^	2.47 ± 2.20
Occlusion 6	3.54 ± 3.26	2.22 ± 1.31	2.27 ± 1.90
Occlusion 7	3.35 ± 2.66	2.11 ± 1.45	2.31 ± 1.83
Occlusion 8	3.84 ± 3.37	2.36 ± 1.99	2.51 ± 3.03
Occlusion 9	3.42 ± 2.49	2.07 ± 1.83	2.48 ± 1.84
Occlusion 10	3.11 ± 2.72^c^	1.83 ± 1.23^b,c,d^	2.23 ± 2.22

There was a significant occlusion effect for SmO_2_ fast desaturation rate (*p* < 0.001; Table 2) and session effect for SmO_2_ fast desaturation rate (*p* = 0.020; Table 2), but no session by occlusion effect (*p* = 0.940). In session 1, FDR was significantly different in occlusions 2, 3, 4, 5, 6, 7, 8, 9, 10 compared to occlusion 1 (Table 2). Occlusion 2 was significantly different from occlusion 3, 4, 5, 6, 7, 8, 9 and 10, occlusion 3 was significantly different from occlusion 4, 5, 6, 7, 8, 9 and 10 (Table 2). Occlusion 4 was significantly different from occlusion 6, 7, 8, 9 and 10, occlusion 5 was significantly different from occlusion 6, 7, 8, 9 and 10 (Table 2). Occlusion 6 was significantly different from occlusion 8, 9 and 10, occlusion 7 was significantly different from occlusion 8 and 10 (Table 2). Occlusions 8 and 9 were significantly different from occlusion 10 (Table 2). In session 2, occlusions 1, 2 and 4 were significantly different from occlusions 6, 7, 8, 9, 10 (Table 2). Occlusion 3 was significantly different from occlusions 7, 8, 9 and 1, occlusions 5, 6, 7 and 8 were significantly different from occlusions 9 and 10 (Table 2). Occlusion 9 was significantly different from occlusion 10 (Table 2). In session 2, occlusions 7, 8, 9 and 10 were significantly different from the same occlusions in session 1. In session 3, occlusion 1 was significantly different from occlusion 3, 4, 7, 8, 9 and 10, occlusion 2 was significantly different from occlusion 6, 7, 8, 9 and 10. In session 3, occlusions 5, 6, 7, 8, 9 and 10 were significantly different from the same occlusions in session 1

^a^*p* < 0.05 compared to occlusion 1

^b^*p* < 0.05 compared to occlusion 2

^c^*p* < 0.05 compared to occlusion 3

^d^*p* < 0.05 compared to occlusion 4

^e^*p* < 0.05 compared to occlusion 5

^f^*p* < 0.05 compared to occlusion 6

^g^*p* < 0.05 compared to occlusion 7

^h^*p* < 0.05 compared to occlusion 8

^i^*p* < 0.05 compared to occlusion 9

*p < 0.05 compared to session 1

## Discussion

The aim of this study was to examine mitochondrial changes and improvements in incremental time to exhaustion over a 3 week period using standard gym ergometers, as opposed to the more costly lab-based alternatives. This is the first paper to demonstrate the effectiveness of repeated sprint training within professional boxing, as well as its effect on rectus femoris oxygenation and mitochondrial function. The rectus femoris plays a crucial role in key boxing activities like movement and generating punch force during a boxing match (Dunn et al. 2020; Lenetsky et al. 2020; Usher and Babraj 2023). Enhancing this muscle’s oxidative capacity is therefore critical, as it enables boxers to maintain consistent performance across multiple rounds, effectively reducing the risk of peripheral fatigue that could lead to a decline in performance. This study presents a promising training intervention due to its flexibility and short duration, allowing it to be periodised easily into a training camp.

### Time to exhaustion

There was a significant increase in incremental treadmill time to exhaustion following 3 weeks of sprint interval training as compared to testing session 1 and 2 but no change across the control period (Fig. 2). Time to exhaustion is an important aspect of incremental exercise testing but the exact reason for failure is not known. In trained runners, incremental time to exhaustion has been shown to increase by 6.4% following 2 weeks of SIT with a 1:3 work to rest ratio (Kavaliauskas, Rodrigo R Aspe and Babraj 2015), which is similar to the size of change seen in session 3 as compared to session 1 or 2 in the current study (Fig. 2). Following SIT, incremental time to exhaustion can be increased with no change in VO_2_ max (Kavaliauskas et al. 2015). When low intensity training is done then incremental TTE is only increased with blood flow restriction (Corvino et al. 2014). Taken together this shows that the point of failure during an incremental test is largely derived from peripheral rather than central adaptation. At the point of failure, the same muscular metabolic disturbance is seen after sprint interval training, although with a much greater workload and with a reduction in central fatigue developed due to a resistance of central drive to the type III/IV afferent signal (O’Leary et al. 2017). At the point of failure, we see a greater smO_2_ in the skeletal muscle as compared to presprint interval training in professional boxers (Table 1). This may reflect a greater type III/IV afferent activity following training which would promote a greater ventilatory reflex response leading to an increased muscle perfusion during higher workloads allowing for a greater sustained effort (Amann 2011). Further, following 7 sessions of sprint interval training consisting of 4–7 30 s sprints, Laursen et al. (2020) report a greater mitochondrial affinity for oxygen suggesting improved efficiency of oxygen use across increasing workloads. This may explain the greater smO_2_ seen that we report at the point of failure (Table 1). More research is needed to determine the underpinning physiological changes that prevent fatigue following sprint interval training, but this adaptation is crucial to allow sustained performance during competition for a professional boxer.

### Mitochondrial rate of change

After 3 weeks of sprint interval training there was a significant increase in mitochondrial rate compared to session 1 or 2, as measured post-volitional exhaustion (Fig. 3). Across the occlusion period heart rate was relatively consistent at all 3 testing sessions, suggesting a similar systemic delivery of blood to the recovering skeletal muscle. This is the first study to utilise recovery mitochondrial rate following volitional exhaustion, all other papers have carried our exercise either at a low percentage of maximum force or following electrical stimulation (Ryan, Brizendine and McCully 2013; Maliszewski et al. 2024). In session 1 and 2 there is no saturation in the monoexponential curve across the occlusions in well trained professional boxers (Fig. 3A, B, Table 2). This is similar to the response seen following electrical stimulation in people with spinal cord injury which is suggested to involve mitochondrial disturbance (Ghatas et al. 2020). Therefore, no saturation in the monoexponential fit may reflect the nature of disturbance in the skeletal muscle at the point of volitional exhaustion with changes in mitochondrial structure, function and energy production suggested as important factors in fatigue development (Filler et al. 2014). Further, to the best of our knowledge this is the first intervention study looking at change in NIRS derived mitochondrial rate following a training intervention, although others have shown a relationship to aerobic fitness (Lagerwaard et al. 2019). Sprint interval training has been shown to change activity levels of mitochondrial enzymes (Macdougall et al. 1998) and increase the rate of mitochondrial protein synthesis post exercise leading to remodelling of mitochondrial function within the skeletal muscle (Scalzo et al. 2014). Further the increased mitochondrial rate function post training may also be related to better oxygen extraction at maximal exercise and during recovery and a lower mitochondrial sensitivity to ADP due to a reduction in p50mito (Larsen et al. 2020) leading to greater mitochondrial affinity for oxygen and faster muscular utilisation of oxygen during recovery. Improvement in oxygen affinity in the mitochondria has been suggested to be controlled by elevated cytochrome c oxidase activity (Larsen et al. 2020) which has been shown to be increased following SIT (Macdougall et al. 1998). In the current study we see an increase in the area under the post-exhaustion SmO_2_ curve (Table 1) which suggests a faster refill of oxygen availability of the skeletal muscle post sprints that would reflect greater efficiency of oxygen use during recovery. As such sprint interval training has been shown to improve PCr recovery kinetics post exercise (Forbes et al. 2008).

### Recovery

Recovery from fatiguing exercise is a crucial component with sporting performance (Mika et al. 2016; Kellmann et al. 2018). In this study the lack of variation in muscle reoxygenation rates suggests a potential disparity between oxygen desaturation and resaturation at the point of voluntary exhaustion. This observation may align with findings that reoxygenation rates can diverge from desaturation and oxygen kinetics after initial sprints (Buchheit and Ufland 2011). Muscle reoxygenation post exercise is influenced by factors like blood flow, the metaboreflex response, and capillary dynamics. Enhanced blood flow and muscle oxidative enzyme activity are linked to better muscle reoxygenation post exercise (Kime et al. 2003; Puente-Maestu et al. 2003). The metaboreflex, particularly in ischemic conditions, boosts arterial haemoglobin, thus improving oxygen delivery (O’Leary et al. 2017). However, when lactate is elevated in muscles or blood, it has been suggested it can limit or restrict post-exercise reoxygenation (McCully et al. 1994). Despite similar levels of metabolic disturbance at the point of failure before and after training, lactate concentrations remain heightened post exhaustion and may be further elevated following interval training (Juel et al. 2004). Consequently, the increased oxygen levels at exhaustion after SIT could obscure observable changes in muscle reoxygenation due to a potentially stronger metaboreflex response. There’s a need for more research to understand SIT’s effects on muscle reoxygenation dynamics.

### Adaptations in well trained athletes

Professional boxers typically engage in a well-established training regimen, which includes technical pad work, heavy bag exercises, strength and conditioning, and sparring. This training process, while standardized, often lacks a structured approach specifically aimed at physiological adaptation (Usher and Babraj 2023). Despite this, boxers are considered well-trained athletes, proficient in their specialised requirements. Similar studies to this one have shown that even well-trained athletes can experience further physiological adaptations from SIT training. For example, in well trained cyclists sprint training increased endurance capacity and time trial performance over 4 weeks. However, intensity is the key determinant of adaptation. In the current study, athletes controlled the intensity based on expected heart rate response to ensure they had the correct intensity on the different cycles used. This may represent a simpler way for athletes to control effort than using VO_2_ max (Laursen and Jenkins 2002) or anaerobic power reserve (Wang and Zhao 2023).

## Limitations

This is a pre and post study of the effects of a three-week repeated sprint intervention with well trainer professional boxers. As such no attempt was made to control the athletes training load before taking part in the study. Repeated sprints were conducted on available gym-based bike ergometers, with all three sprint times decided by the athlete as per the study design. This may have affected the magnitude of adaptation, although heart rate data was utilised to gauge and guide the repeated sprint intensity levels. No attempt was made to monitor the athlete diets, any changes in diet over the course of the study may have the potential to alter mitochondrial function (Putti et al. 2015). However, athletes were told to maintain normal diet and it has been shown that people rarely change their diet (Pai and Sabharwal 2022) but future research should look to record dietary habits.

## Perspectives

This research is the first to investigate the effects of SIT in professional boxers and on noninvasive of mitochondrial rate changes post volition. There is significant performance and physiological adaptations after SIT, demonstrating it is an effective training protocol for professional boxing. The study demonstrates increased incremental time to exhaustion and higher SmO_2_ at volitional fatigue, indicating improved oxygen affinity and use within the muscle. We also report increased mitochondrial rates post-training like those shown from biopsy (Holloszy 1982; Brizendine et al. 2013; Sumner et al. 2020). Together they highlight the importance of SIT for boxing, allowing greater endurance to allow the athlete to perform consistently across rounds and fatigue resistance will improve cognitive readiness (Aidman 2020) allowing the athlete to be safer in the ring. Further, SIT can be performed on any standard gym bike if the athlete knows the intensity that must be utilised. In the current study heart rate was used as a method to ensure intensity is met with athletes reaching 160 bpm in the first sprint. This is easier for an athlete or coach to implement than other approaches. Taken together we show the effectiveness of a short-term intervention for professional boxers, but more research is needed to determine optimal protocols and maximise adaptation.

## Supplementary Information

Below is the link to the electronic supplementary material.

Supplementary file1 (DOCX 16 KB)

## Data Availability

Data will be available upon reasonable request.
