# Supplementary material for: Impact of sprint interval training on post-fatigue mitochondrial rate in professional boxers
Source: Eur J Appl Physiol. 2024 Sep 3;125(1):261–71. doi: 10.1007/s00421-024-05594-0 (PMC11747053; doi:10.1007/s00421-024-05594-0)
Supplement: Supplementary file 1 — Supplementary file1 (DOCX 16 KB) [file 421_2024_5594_MOESM1_ESM.docx]

Table S1: Maximum and minimum SmO_2_ during post exercise occlusions

|  | **Session 1** | **Session 2** | **Session 3** |
| --- | --- | --- | --- |
| **Max SmO_2_** |  |  |  |
| Occlusion 1 | 79.1 ± 10.0 | 81.2 ± 8.8 | 81.0 ± 8.9 |
| Occlusion 2 | 87.9 ± 7.9^a^ | 85.9 ± 7.9^a^ | 89.6 ± 5.9^a^ |
| Occlusion 3 | 87.6 ± 9.4^a^ | 87.7 ± 9.3^a, b^ | 89.2 ± 8.2^a^ |
| Occlusion 4 | 86.9 ± 8.0^a^ | 89.3 ± 7.3^a, b^ | 90.5 ± 6.5^a^ |
| Occlusion 5 | 84.4 ± 6.9^b, c, d^ | 87.7 ± 8.0^a^ | 88.6 ± 8.9^a^ |
| Occlusion 6 | 83.1 ± 6.1^b, c, d^ | 88.0 ± 7.4^a^ | 88.6 ± 8.1^a^ |
| Occlusion 7 | 83.3 ± 7.4^b, c, d^ | 85.9 ± 8.1^d, e^ | 87.3 ± 8.2^a^ |
| Occlusion 8 | 82.0 ± 7.9^b, c, d^ | 85.5 ± 9.0^d, f^ | 86.6 ± 6.2 |
| Occlusion 9 | 82.1 ± 7.1^b, c, d^ | 85.7 ± 7.6 | 83.5 ± 7.9^d, f, g, h^ |
| Occlusion 10 | 80.6 ± 6.2^b, c, d, e, f, g^ | 83.7 ± 8.6^i^ | 83.3 ± 5.9^b, d, f, g^ |
|  |  |  |  |
| **Min SmO_2_** |  |  |  |
| Occlusion 1 | 47.8 ± 18.8 | 60.8 ± 13.4^*^ | 54.8 ± 19.2 |
| Occlusion 2 | 51.6 ± 12.4 | 62.5 ± 15.6^*^ | 62.2 ± 15.7^*a^ |
| Occlusion 3 | 53.9 ± 7.5 | 63.3 ± 17.3 | 63.1 ± 14.1^*a^ |
| Occlusion 4 | 57.0 ± 6.4^c^ | 64.4 ± 15.9 | 66.5 ± 13.5^*a, b, c^ |
| Occlusion 5 | 56.2 ± 7.7 | 64.9 ± 17.8 | 64.6 ± 13.9^a^ |
| Occlusion 6 | 55.3 ± 7.9 | 65.3 ± 15.6^*^ | 66.5 ± 11.3^*a, c^ |
| Occlusion 7 | 53.1 ± 9.1 | 65.9 ± 14.7 | 66.0 ± 10.0^*a^ |
| Occlusion 8 | 54.7 ± 9.0^g^ | 62.7 ± 22.8 | 65.6 ± 8.6^*^ |
| Occlusion 9 | 55.1 ± 9.5 | 65.5 ± 16.6 | 64.0 ± 7.9 |
| Occlusion 10 | 58.3 ± 6.9^f, g, h^ | 65.8 ± 15.6 | 63.6 ± 7.3 |

a=p<0.05 compared to occlusion 1; b=p<0.05 compared to occlusion 2; c=p<0.05 compared to occlusion 3; d=p<0.05 compared to occlusion 4; e=p<0.05 compared to occlusion 5; f=p<0.05 compared to occlusion 6; g=p<0.05 compared to occlusion 7; h=p<0.05 compared to occlusion 8; i=p<0.05 compared to occlusion 9; * =p<0.05 compared to session 1
